# Friction ridge analysis in disaster victim identification (DVI): Brazilian case studies

**DOI:** 10.1080/20961790.2021.1882745

**Published:** 2021-04-08

**Authors:** Marco Antonio de Souza, Gabriel de Oliveira Urtiaga, Renata Cristina Grangeiro Ferreira, Luciene Marques da Silva, Jade Kende Gonçalves Umbelino, Flávio Roberto de Melo, Simone de Jesus

**Affiliations:** a Instituto Nacional de Identificação, Polícia Federal, Brasília, Brazil; b Grupo de Identificação, Superintendência Regional da Polícia Federal, Porto Alegre, Brazil; c Departamento de Polícia Técnica, Polícia Civil do Distrito Federal, Brasília, Brazil; d Núcleo de Identificação, Superintendência Regional de Polícia Federal, Belo Horizonte, Brazil; e Instituto de Identificação, Polícia Civil, Goiânia, Brazil

**Keywords:** Forensic sciences, disaster victim identification, DVI, fingerprint, ridgeology, Alethia System

## Abstract

Depending on the magnitude and nature of a disaster, identifying the victims can be a complex task that requires coordinated work by disaster victim identification (DVI) teams based on pre-established protocols. Thus, the analysis of fingerprints has been presented as a method to establish, when possible, the identity of the victims during the DVI process. This study discusses the importance of this primary method of identification and the results obtained in four different disasters in which Brazilian DVI teams were involved: the Air France Flight AF447 plane crash in the Atlantic Ocean, floods and mudslides in the State of Rio de Janeiro, Brazil, the LaMia Flight 2933 plane crash in Colombia, and the tailings dam collapse in Brumadinho, Brazil. Here, we also report the use of the automatic fingerprint capture and identification system, called Alethia, developed by the Federal Police of Brazil and used in the victim identification process in the two latter events mentioned above.

**Key points**
- This article presents four different disasters that occurred in Brazil and overseas and involved Brazilian DVI teams in the identification process, focusing on fingerprint identification (Air France Flight AF447, floods and mudslides in the State of Rio de Janeiro, Brazil, LaMia Flight 2933, and the Brumadinho tailings dam collapse).
- This article also describes the evolution of the DVI process in Brazil, including a description of the technology currently used by Brazilian fingerprint experts (Alethia).
- This article reports how the Alethia System was used in the disasters and how it optimized the human identification process when compared to traditional methods.

## Introduction

Several studies on procedures adopted for disaster victim identification (DVI) can be found in the literature [1–4]. A massive disaster is defined as an unexpected event that causes the death of many people. These events may be natural disasters, terrorist attacks, massive car accidents, plane crashes, and explosions, among others [5].

Disaster events can be classified as open or closed. An open disaster is characterized as an unexpected event that results in the death of several unknown individuals in which qualitative or quantitative information about the fatal victims is not available and further investigation is needed (an earthquake, for example). However, a closed disaster has a fixed number of victims whose information is already known, which enables their identification (such as a plane crash) [5].

To establish baseline standards for DVI operations, the International Criminal Police Organization (INTERPOL) published the first guide to DVI in 1984. Since then, it has been revised over the years. The INTERPOL DVI Guide provides guidelines for the management of DVI operations through synchronization of diplomatic, political, and law enforcement actions, especially in multinational operations [5].

DVI teams are formed by experts in different areas who work collaboratively to identify victims. The coordination of the DVI work begins as soon as a disaster occurs, which is important for an effective response. The top priorities in disaster responses are rescuing survivors and minimizing loss of life [5].

Immediately after the disaster, the lead authority (the police or others depending on the region and jurisdiction) assumes the command responsibility for the disaster and makes the first decisions that will influence the next phases of the DVI process. They are responsible for analysing the situation with respect to the type of disaster, access to the area, estimated number of victims, acquisition of evidential material, and other logistical issues [2, 5].

According to the INTERPOL DVI Guide, the DVI phases are as follows: (1) scene; (2) postmortem (PM); (3) antemortem (AM); and (4) reconciliation [5]. The scene must be treated as a crime scene and all human remains, exhibits, and property should be left at the location until the scene examiners complete their work. All human remains recovered from the scene must be processed, examined, and stored in a mortuary or location tempora­rily built for the operation. The identification is made by compiling and analyzing all data obtained in the PM and AM phases. PM examinations, conducted by PM teams, include photographs, friction ridge analysis, comparative dental analysis, DNA analysis, and autopsy. The AM information comprises data collection about the missing persons, family, relative, or friend interviews to obtain sufficient facts, dental and medical record searches, fingerprint collection, DNA analysis, documents, photographs, and any other data relevant to identifying the victims. During the reconciliation phase, investigators are responsible for matching PM with AM data to identify the victims. Once identification is successful, arrangements are made to return all human remains to the respective family [2, 5].

The methods associated with the identification process are classified as either primary or secondary. The primary methods are friction ridge, comparative dental, and DNA analyses. Serial numbers from medical implants can also be used as a primary method. Other procedures, such as personal descriptions, tattoos, medi­cal findings, among others, are classified as secondary methods [5]. Friction ridge analysis is considered the fastest primary identification method [6].

DVI teams are composed of a multidisciplinary technical staff that includes fingerprint experts. To make friction ridge analysis feasible, rescue teams should immediately evaluate the preservation and safeguard of the victims’ hands, then determine the best fingerprint collecting method considering the hands’ conditions. Should collecting this kind of evidence not be possible or should there be no match between PM and AM data, the identification must be made by other methods such as dental or DNA analyses [7].

This study discusses the work of Brazilian DVI teams in four different disasters that occurred in Brazil and overseas with specific focus on friction ridge analysis. The disasters include the crash of the Air France Flight AF447 in the Atlantic Ocean, floods and mudslides in the State of Rio de Janeiro, Brazil, the crash of the LaMia Flight 2933 in Colombia, and the Brumadinho tailings dam collapse in the State of Minas Gerais, Brazil. This paper also addresses the evolution of the techniques adopted by Brazilian fingerprint experts, which currently include the use of digital fingerprint capture technology, the Alethia System.

**Figure 1.**
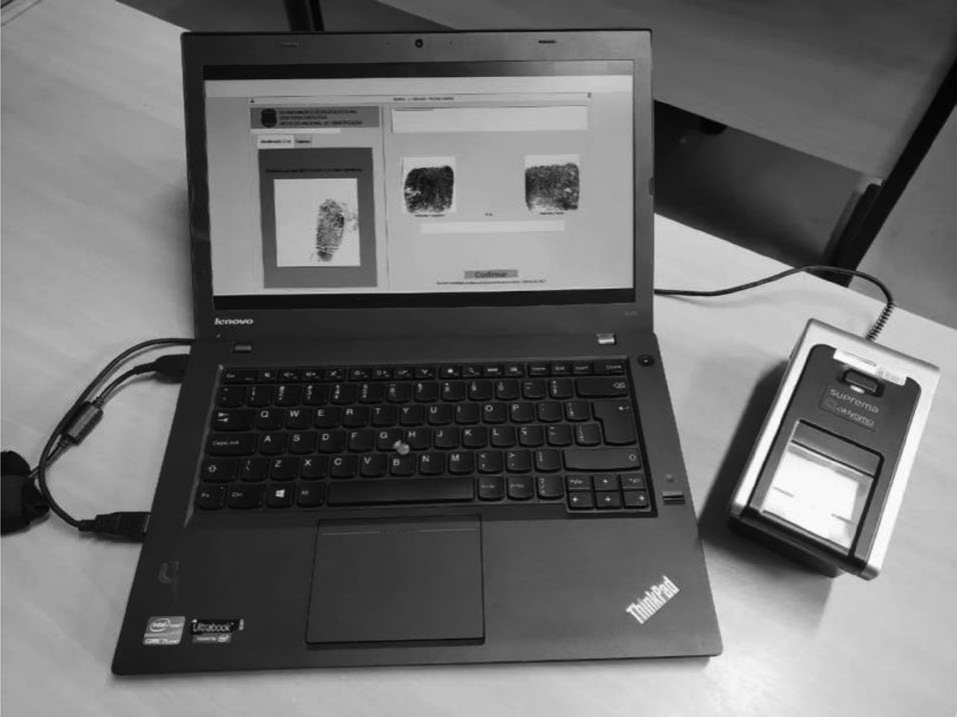
The Alethia System used by the Federal Police of Brazil.

The Alethia System (Figure 1) is a biometric scanner connected to a portable Automated Fingerprint Identification System (AFIS) developed by the National Institute of Identification (of the Federal Police of Brazil —INI/PF). The system has remote access to detailed friction ridge information that was previously collected. This information is used to optimize the DVI process. The crash of the LaMia Flight 2933 was the first time that this system was ever used in such a process by Brazilian experts.

## Methodology

All data presented in this paper were obtained from records of the Federal Police of Brazil. The discussion below is focused on the use of friction ridge analysis in the DVI process. For each disaster, a brief description is provided, as well as the place and nature of the incident, the number of victims, the degree of fragmentation and decomposition of the bodies, and whether AM data were available or not.

## Results

### Air France Flight AF447

On the night of May 31, 2009, the Air France Flight AF447 crashed into the Atlantic Ocean en route from Rio de Janeiro to Paris with 228 people onboard (216 passengers and 12 crew members of 33 nationalities), 59 of which were Brazilians. According to the International Civil Aviation Convention, the investigations were conducted by the French government, as the aircraft was French. Brazil was responsible for the search and identification of the victims and for assisting with the collection of the wreckage. One week after the accident, Brazilian and French rescue teams had recovered 50 bodies and 10% of the plane, an Airbus A330 [8].

The first stage of PM trace collection was carried out on the island of Fernando de Noronha, located on the Brazilian northeastern coast, where a precursor team had built a field mortuary to record belongings and collect fingerprint samples and biological material for DNA ana­lysis. Autopsies and dental analysis were performed at the local mortuary in Recife, the capital of the state of Pernambuco. A crisis committee and reconciliation centre were created for AM and PM data collection. DNA tests were performed in Brasília. Teams from several countries worked on the search for AM data. In Brazil, the headquarters of Brazilian AM teams was located in Rio de Janeiro. Characterized as a closed disaster, the victims’ data were obtained from the Brazilian Government’s Civil Database and INTERPOL. Fingerprint data were compared using the AFIS, used by the Federal Police of Brazil.

The first bodies were found on June 6, 2009, and in the first stage of the search, 50 bodies were recovered: 20 Brazilians (12 men and 8 women) and 30 foreigners (13 men and 17 women). On August 6, the identification work was completed with the last victim having been identified. The recovered body remains showed stages of advanced decomposition with multiple fractures and mutilations. Because of the deteriorating condition of the epidermis, finger skin was removed by degloving and placed on the fingertip of one of forensic technician. In some cases, the fingers were put through a drying process and, soon after, through a micro-adhesion technique. This consisted of brushing regular black powder onto the fingers, followed by collecting the fingerprints on an adhesive support. Another technique used to collect fingerprints was direct photography of friction ridges that had been enhanced after sprinkling on regular black powder. The friction ridge analysis managed to identify 14 victims (28.0%) of the 50 bodies recovered in the first stage of the search.

### Floods and mudslides in the State of Rio de Janeiro

Because of the large volume of rainfall that occurred in the mountainous region of the State of Rio de Janeiro in January 2011, some cities, especially Petrópolis, Teresópolis, and Nova Friburgo, were ravaged by heavy floods and mudslides that caused great social damage and led to the death of 895 victims [9]. It was a traditional open disaster where identification was a great challenge because members of many families died and, in some cases, there were no relatives left.

The multidisciplinary DVI team, composed of experts in human identification, was sent to the locations and formed a task force. The team was divided into two groups: one was sent to Teresópolis and the other to Nova Friburgo. These cities were the most affected by the tragedy and had a higher number of fatalities.

The physical structure of Nova Friburgo Institute of Education was temporarily used as a mortuary to receive the victims’ bodies and collect PM data. Fingerprints and DNA were collected and dental analyses and autopsies were performed. Some classrooms were used as a centre for the victims’ families and friends where they offered notarial services, public defenders, prosecutors, psychological support, and funeral services. This dynamic helped reduce the excessive and time-consuming administrative procedures followed to release the bodies so the families could perform their funeral rites.

The bodies were numbered at the entrance of the gym with a tag. They were arranged on stretchers and a PM number was assigned. Then, photographic records were taken of each victim in the frontal and dorsal decubitus positions and of their robes and belongings. All information extracted from the bodies was gathered in reports with the same number assigned to the corpse and forwarded to the sector responsible for compiling the data and analyzing whether there is any match or not. The AM fingerprint data were obtained by the Félix Pacheco Institute of Identification in Rio de Janeiro, where the reconciliation process also took place.

The bodies were in an early state of decomposition, with most of them in a state of maceration. Before collecting their fingerprints, their hands were cleaned with soap and water, dried with paper towels, and isopropyl alcohol was applied. In cases in which the epidermis was intact, the conventional method was adopted for recording the fingerprints, consisting of applying a thin layer of black printer ink on the fingers and then recording the friction ridge impressions onto a fingerprint card. In cases in which there was only the dermis left (severely damaged or absent epidermis), the micro-adhesion technique was used.

The bodies examined by the team assigned to work in Nova Friburgo were in a sufficient condition for fingerprint collection. They had excellent visualization of the friction ridges and good comparison conditions, despite the early state of putrefaction. Out of the 895 bodies registered, 527 were identified. Fingerprint ana­lysis accounted for the identification of 487 victims (92.4%). Other methods, such as DNA and dental ana­lyses, together identified 40 victims (7.6%).

### LaMia Flight 2933

On November 28, 2016, the LaMia Flight 2933 crashed near Medellín, Colombia, killing 71 people of the 77 onboard. The aircraft was transporting the Brazilian Chapecoense football team and associates from Santa Cruz de la Sierra, Bolivia to Medellín, Colombia, where the team was scheduled to play the 2016 South American Football Cup Finals. This was a charter flight operated by Linea Aérea Merideña Internacional de Aviación, identified as LMI2933, at the service of the Chapecoense Football Association.

There were 73 passengers and four crew members onboard. The crew consisted of a commander, co-pilot, and two cabin crew members. One of the four crew members, three of the football players, and two other passengers survived the crash with injuries [10].

A few hours after the crash, a search and rescue ­operation was launched by first responders from the Colombia Air Force. The bodies of all 71 victims were taken to the mortuary in Medellín (National Institute of Legal Medicine and Forensic Sciences — NILM/Medellín) for identification.

The DVI operation started by collecting AM information about the victims by the Brazilian DVI team. The information was available at the AFIS of INI/PF. The files were uploaded to the Alethia System and would later be used by the PM team.

The Alethia database was loaded with fingerprint patterns of the declared victims. In real time, those victims that had not been directly identified by the Alethia AFIS had their friction ridge details accessed remotely and added to Brazil’s national AFIS. The biometric scanner uses optical technology, which enables the collection of friction ridge information from the victims’ bodies [6].

The PM team carried out their activities at the NILM/Medellín where, in addition to the fingerprints of the victims, the reconciliation phase began. The bodies were sent by rescue teams to the NILM/Medellín in numbered body bags. Then, each victim’s face was photographed. The state of conservation of the bodies indicated that the deaths had recently occurred as they did not show the typical signs of putrefaction. Moreover, although most bodies had blunt wounds and/or fractures, little damage was observed in the victims’ external tissues (dermis and epidermis). There was no process of fragmentation of limbs among the bodi­es of the victims later identified as Brazilians, except in one victim. Before Brazilian experts collected the Brazilian victims’ fingerprints using the Alethia System, the hands of the victims were cleaned with water. If the victim’s identity was found in the system, the screen containing the identification information would be photographed. If there was not any match, attempts were made toward skin reconstitution by injecting glycerine [11] or boiling treatment [12]. Then, fingerprints were collected once more using the biometric reader and, in case of another negative result, ink and a fingerprint card were used.

All the perfect matches using the Alethia System were sent to the reconciliation centre in Brazil, where the veri­fication examination was performed, and the respective Identification Report was issued. For the cases not identified through the Alethia System, the PM prints collected in the NILM/Medellín were compared with the AM documents from passport databases and collection forms for civil identity registration of state security secretariats. The Colombian Team, however, had applied the traditional thin layer of black printer ink to the Colombian victims’ fingers and recorded the friction ridge impressions onto a fingerprint card.

All 64 Brazilian victims of this air accident were identified by fingerprint analysis. Out of these 64 individuals, 52 had been previously identified by the Alethia System. These identifications were verified and confirmed at the Reconciliation Centre. The remaining 12 identifications that had not been previously made by the Alethia System, because of the low quality of the fingerprints or absence of standard material in the Alethia System database, were confirmed and verified directly in the national AFIS.

The use of a portable database along with a biometric reader, such as the Alethia System, stands out as an essential tool for optimizing DVI. Given the high number of countries that adopt fingerprint databases for civil and criminal identification purposes, this methodology is appropriate to identify victims of air disasters.

### Brumadinho tailings dam collapse

On January 25, 2019, a dam owned by the mining company Vale S/A collapsed in Brumadinho, Brazil, dumping 0.012 km3 of mineral tailings over an area of approximately 2.9 km2. Brumadinho is a town located about 50 km from Belo Horizonte, the capital of the state of Minas Gerais, Brazil. Rescue teams searched for missing persons until March 21, 2020, 421 days after the event. Overall, the event killed 270 people [13]. The searches ceased because of the COVID-19 state of emergency declared by the Minas Gerais state government. The mud and its mineral composition seemed to have produced sufficient environmental conditions to conserve the victims’ bodies. This is one of the largest environmental disasters and the largest occupational accident in Brazilian history, as well as one of the largest ore dam collapse disasters in the world [14].

The victim identification process was carried out by the Minas Gerais State Department of Public Security, with support from the Federal District Civil Police and Federal Police of Brazil. The procedures related to the primary identification methods took place at the headquarters of the Federal Police of Brazil located in Minas Gerais (AM teams) and in the mortuary of Belo Horizonte (PM teams). The AM teams searched for the fingerprint files of the individuals declared missing by their families. These teams also converted the files into National Institute of Standards and Technology (NIST) file extension and uploaded them into the Alethia System. The NIST file extension defines fingerprint image quality.

Once located, the body or human remains were transferred to the mortuary of Belo Horizonte, Brazil. The PM team initiated the registration with photos and descriptive forms. The next step involved the friction ridge collection. Handwashing was performed and segment conditions were assessed, at which time the most appropriate treatment or technique was determined (black inking, micro-adhesion, degloving, boiling treatment, silicone use, direct photography, and so on). The fingerprints were subsequently collected using the Alethia System.

The victims’ bodies were found in several decomposition states, most of them in maceration process and some with evidence of mummification. To date, 270 missing persons have been reported, including 261 (96.7%) already identified by DVI teams. Fingerprint analysis accounted for 195 (74.7%) identifications. The other identifications were made by dental and DNA analyses and forensic anthropology teams.

Considering the 185 identifications made between January 28 and February 26, 2019 (the time interval during which the Alethia System was used), 29.7% of them were made directly through Alethia. In addition, the remote connection allowed 17.3% of the identifications to be made through Brazil’s national AFIS by real-time remote access to the friction ridge details *via* Alethia. The Alethia System contributed directly or indirectly to the identification of 47.0% of the victims, making the DVI process faster and more optimized. The observed results indicate that the use of the Alethia System in DVI is advantageous because it enables the immediate communication of the collected data to remote teams through the use of computer networks and speeds up the identification procedure, reducing the suffering of the families involved in tragedies.

The victims not recognized by Alethia can be explained by the absence of the fingerprint patterns in the AFIS, as the database had been updated gradually as missing persons were being reported to the authorities, or by the poor quality of some fingerprints provided by regional institutes of identification, some of them without AFIS. Therefore, the fingerprints not recognized can be a limitation to the friction ridge analysis. The last identification made by fingerprint analysis was possible by micro-adhesion technique followed by direct photo­graphy as much as 88 days after the disaster.

## Discussion

The identification of victims in cases of mass disasters is one of the most important tasks following the event. Many steps have recently been taken in Brazil with respect to this. The DVI INTERPOL Guide was used as a model for developing the DVI manuals that the Federal Police of Brazil and the state police use.

The case of floods and mudslides in the State of Rio de Janeiro, an open disaster [5], had a total victim identification rate of 58.9%, whereas 100% of the victims were identified in the LaMia Flight 2933 disaster. The nature of the event, whether open or closed, demonstrated to be an important factor in the identification process. In the case of the Brumadinho tailings dam collapse, most of the victims were employees of company Vale S/A. This enabled the identification of 96.7% of the victims because there was significant information available about the victims involved in the tragedy.

In disasters in which the victims were from various countries, the participation of experts from all nations involved should be ensured to facilitate information exchange and data acquisition [5]. Certainly, the participation of Brazilian DVI teams during the identification of the victims of the LaMia Flight 2933 optimized the identification process. Table 1 summarizes all the case studies presented here.

**Table 1.** Case studies.

Disaster victim identification event	Main print development technique	Number of victims	Number of victims identified by friction ridge analysis	Degree of body fragmentation and decomposition	Access to antemortem data
Air disaster— Air France Flight AF447	Black powder	228	14 (28.0%)	Advanced decomposition with multiple fractures and mutilations	Brazilian governmental civil database and International Criminal Police Organization
Floods and mudslides in the State of Rio de Janeiro	Black printer ink	895	487 (54.4%)	Early state of decomposition, most of bodies in a state of maceration	Félix Pacheco Institute of Identification
Air disaster — LaMia Flight 2933	Alethia System	71	64 (90.1%)	Early state of decomposition, blunt wounds and/or fractures	National Institute of Identification
Brumadinho tailings dam collapse	Alethia System, micro-adhesion technique	270	195 (72.2%)	Maceration, mummification, with multiple fractures and mutilations	Federal Police of Brazil in Minas Gerais

The condition of human remains influences the nature and quality of PM data. It also determines which identification method can be used. Although the use of friction ridge analysis is considered the fastest primary method [6], it is important to note that it depends on the condition of the bodies, specifically the palms and fingers. For DVI events that result in partial fragmentation of human remains, burn victims, or underwater conditions, the use of fingerprints for identification can be challenging [15].

The integrity of the body determines whether fingerprints can be collected. Soon after death, the body begins the physical alteration process of its constitution, which has several stages. Among the cadaveric phenomena, the transformative processes are categorized as destructive or conservative. The former includes autolysis, putrefaction, and maceration. Environmental conditions can speed up or slow down these transformative processes. The latter includes saponification, mummification, and calcification [16].

The use of dental analysis presumes a prior know­ledge of the missing person’s identity. Additionally, DNA samples often require genetic profiles of family members. This is the reason why more than one primary method is commonly used in the same investigation to seek victim identification.

Factors such as time and environmental conditions were crucial for the feasibility of applying the friction ridge analysis methodology in the cases presented here. In the disaster involving the Air France Flight AF447, although 50 bodies had been found in the first week after the accident, they were underwater and quickly reached an advanced stage of decomposition. This enabled fingerprint identification of only 28% of the victims. In contrast, the LaMia Flight 2933 crashed on land, so 100% of the identifications were made using victim fingerprints.

In the case of the Brumadinho tailings dam collapse, other factors influenced the work of fingerprint experts, such as the rescue time and the condition of the mud. Despite the remarkable state of maceration seen as time went by, the friction ridges remained relatively conserved, which enabled the identification of the bodies for quite some time after the disaster. Although the searches had been extended for months, it was possible to identify a victim 88 days after the disaster due the conservation state of the bodies, which was hypothesized to be facilitated by the mud.

**Figure 2.**
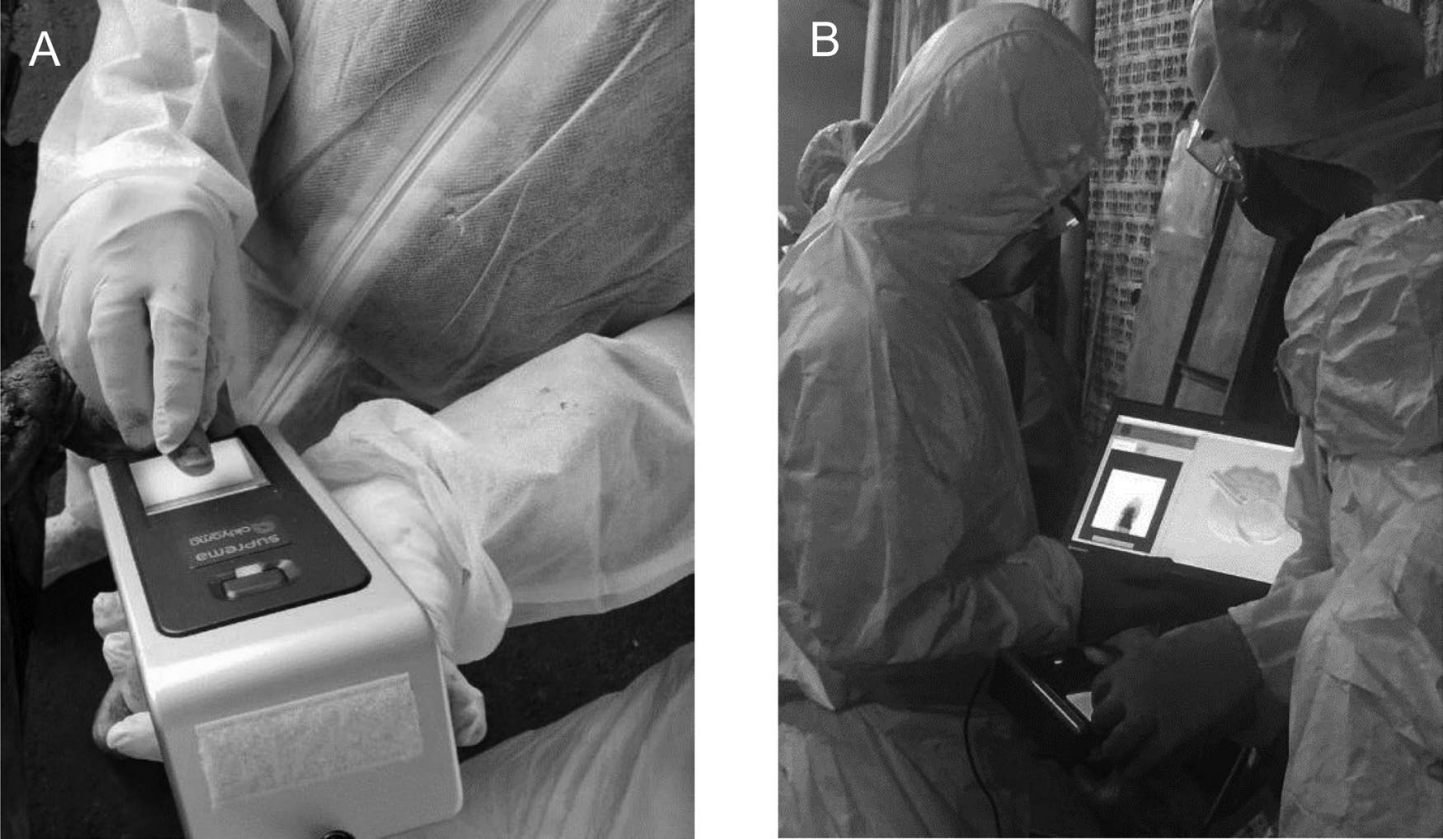
The collection (A) and matching (B) of fingerprints using the Alethia System.

The Alethia System optimized the human identification process in disasters. When up to 60 000 people are registered in the database, it takes, on average, two seconds to identify a victim through fingerprint collection and matching. However, traditional methods using AFIS take several minutes or hours. Figure 2 shows the identification made by the Alethia System, including the fingerprint collection Figure 2A and matching Figure 2B.

The consolidated civil database of fingerprints in Brazil contributes to the ability of DVI teams to identify Brazilian victims by fingerprint analysis. It makes the identification process more efficient when compared with other identification methods and also facilitates the obtention of fingerprint records by AM teams [17]. Biometric databases using fingerprints for visas and passports are growing worldwide. These, and other fingerprint databases, can also be used in a DVI process. As biometrics expands worldwide, obtaining AM fingerprint records of an individual has become easier and faster.

## Conclusion

As the fingerprints of the Brazilian population are regi­stered in a civil database, identification using this primary method is the fastest and most advantageous. As showed here, the Alethia System performed very well when used in DVI events by increasing the number of rapid identifications. Its main benefits include the direct digital collection of the fingerprint without any pre-treatment, such as chemicals or powders, and the immediate comparison with the database.

The decision to use biometric fingerprint devices, like the Alethia System, assumes that the fingerprint patterns available must be of sufficient technical quality (appropriate image resolution, not blurry) to achieve efficient victim identification. In the Brumadinho dam collapse, some data provided by regional institutes of identification affected the identification process by fingerprint analysis.

## Authors’ contributions

Marco Antonio de Souza conceived the study and participated in its design and coordination and drafted the manuscript; Gabriel de Oliveira Urtiaga collected the data and performed the statistical analysis of the Brumadinho tailings dam collapse; Renata Cristina Grangeiro Ferreira collected the data and performed the statistical analysis of the LaMia Flight 2933; Luciene Marques da Silva collected the data and performed the statistical analysis of the Air France Flight AF447; Jade Kende Gonçalves Umbelino collected the data and performed the statistical analysis of the LaMia Flight 2933; Flávio Roberto de Melo collected the data and performed the statistical analysis of the Brumadinho tailings dam collapse; Simone de Jesus collected the data and performed the statistical analysis of floods and mudslides in the State of Rio de Janeiro. All authors contributed to the final text and approved it.
